# A New Technological Approach to Robotic Telesurgery with Starlink: Safe Telesurgery

**DOI:** 10.1016/j.atssr.2025.04.018

**Published:** 2025-05-15

**Authors:** Yuichiro Ueda, Takahiro Kanno, Toshihiko Sato

**Affiliations:** 1 Department of General Thoracic Surgery, Breast and Pediatric Surgery, Fukuoka University School of Medicine, Fukuoka, Japan; 2 Riverfield Inc, Tokyo, Japan

## Abstract

**Purpose:**

This study aimed to perform the world’s first robot-assisted telesurgery for lung resection in an animal model using Starlink (SpaceX) to address cost and latency issues associated with conventional telesurgery communication methods.

**Description:**

A Saroa (Riverfield Inc) surgical robot with haptic feedback function was used in a swine model. The animal was located 1000 km away in Fukushima, Japan, while the surgeon console was located in Fukuoka, Japan. Starlink provided real-time video and data communication. Surgical parameters including safety, latency, and cost were evaluated.

**Evaluation:**

The surgery was completed in 2 hours 44 minutes. Average communication latency was approximately 130 milliseconds, with minor image disturbances occurring once every 5 minutes, potentially due to satellite switching or weather conditions. Despite these interruptions, the surgery was conducted safely.

**Conclusions:**

The successful performance of this telesurgery highlights the potential of low-cost and low-latency satellite communication systems to overcome barriers in telesurgery. These findings pave the way for broader telesurgery applications, particularly in underserved regions, and set the stage for further technical and clinical advancements in remote robotic surgery.

## Visual Abstract

**Figure:**
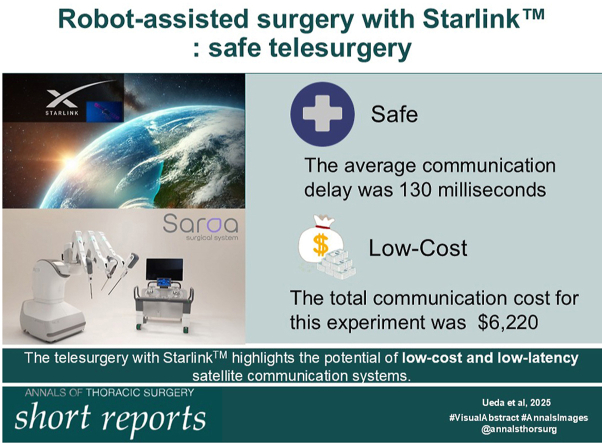

## Technology

Robot-assisted surgery is a procedure in which the surgeon controls a robot while located away from the patient. As robot-assisted surgery has become more common, the possibility of telesurgery using a robot has been discussed. Recent improvements in communication technology have led to exploration of telesurgery using international submarine cables, 5G lines,^1^^,^^2^ and geostationary earth orbit satellites.^3^ However, these technologies have not been used in general yet due to several issues.

One issue is cost. The Lindbergh operation, which was the world’s first telesurgery, conducted in 2001, used the best dedicated line at the time, with an estimated communication delay of 150 milliseconds between Strasbourg, France, and New York, USA. However, this achievement only demonstrates that it is technically possible, and telesurgery using a dedicated line has never been widely performed because of the enormous cost.^4^

To cut communication costs, we chose Starlink in this experiment. Starlink is a satellite internet constellation project developed by SpaceX. The primary goal of Starlink is to provide global high-speed internet access, especially in rural and remote areas where conventional internet infrastructure is limited or nonexistent. The system operates through a large network of low earth orbit satellites. Starlink satellites orbit much closer to the Earth (about 550 km above) than geostationary earth orbit satellites. This allows faster data transmission and lower latency, making this system suitable for a wide range of internet activities.

In the present study, we performed a novel robot-assisted telesurgery of lung resection in an animal model using Starlink.

## Technique

### Robotic System and Network Connections

**Figure 1:**
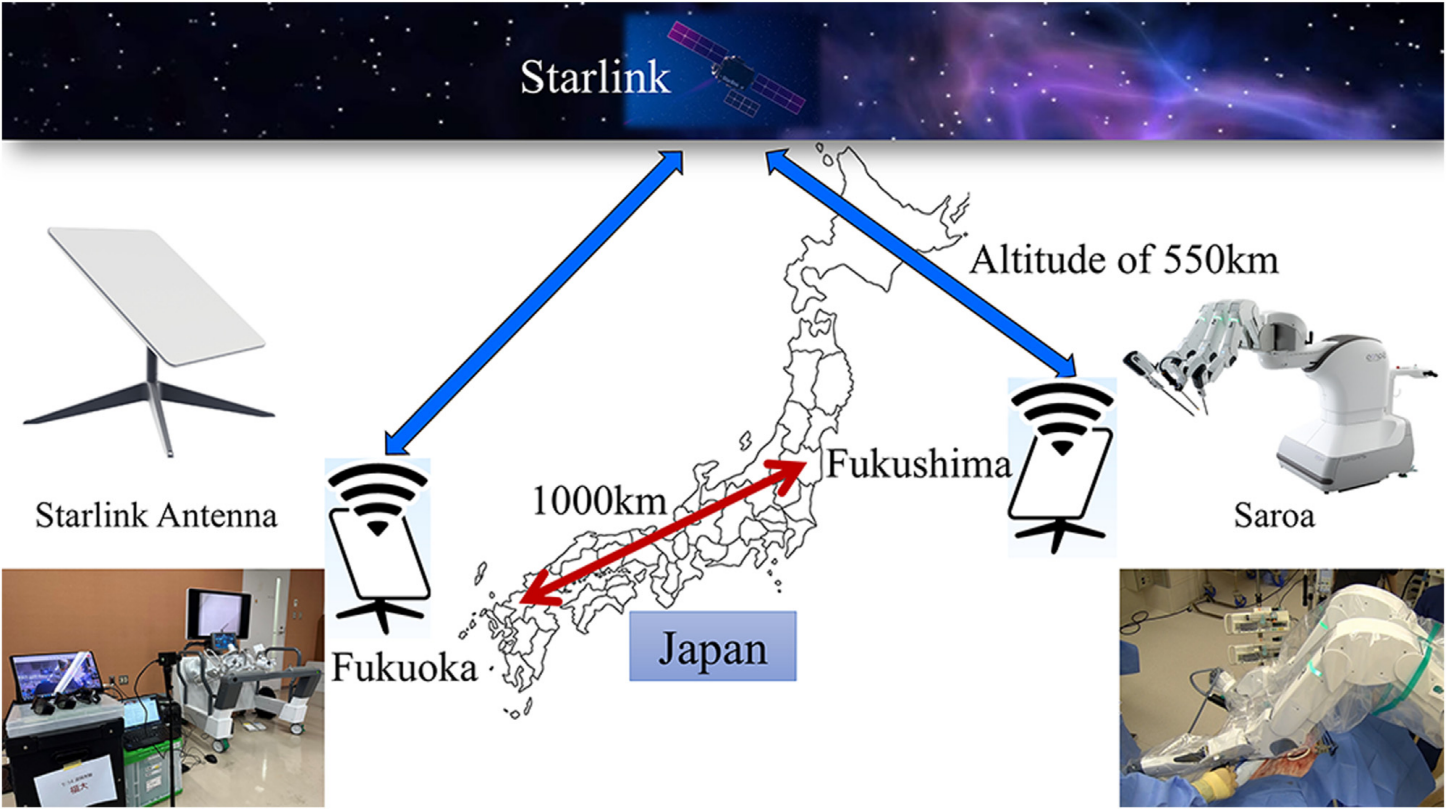
Illustration of the telesurgery experiment.

In this experiment, a Saroa surgical system (Riverfield Inc) was utilized. Saroa is a pneumatically driven robot that provides real-time haptic feedback to the surgeon. We have shown that this robotic system may help to improve surgical safety by facilitating precise procedures that are gentler on tissues.^5^ The patient cart was installed at Fukushima Medical Device Development Support Centre, 1000 km away from Fukuoka, Japan, and the surgeon console was installed in a conference room at Fukuoka University Hospital (Figure 1). The communication system used the Starlink business model, which allowed real-time surgical video and sound confirmation between Fukushima and Fukuoka, and communication between the animal-side surgeon and console surgeon as appropriate. The patient cart sends its timestamp to the surgeon console, and the surgeon console sends back the value. By comparing the console's latest local time and the received reflected timestamp, the round-trip delay can be detected. The latency of the Starlink network was measured using this method.

### Experimental Animal

A healthy swine weighing approximately 60 kg was used in the experiment. The animal received humane care in compliance with the Guide for the Care and Use of Laboratory Animals published by the National Institutes of Health (NIH Publication No. 85-23, revised 1985). All protocols and procedures were approved by the institutional review board of Fukuoka University Hospital (approval no. U23-06-008) and the Fukushima Medical Device Development Support Centre Animal Experimental Committee (approval no. An0000093).

## Clinical Experience

Midazolam (0.1-0.5 mg/kg) and medetomidine (0.02-0.04 mg/kg) were administered intramuscularly to sedate the swine and then isoflurane was inhaled via a mask to induce anesthesia. Mechanical ventilation was maintained using inhalational isoflurane. The swine was placed in the right lateral position under general anesthesia and the Saroa was docked. A left upper lobe anterior segmentectomy was performed by remote control from Fukuoka, with dissection of the pulmonary artery, pulmonary vein, and bronchus (Supplemental Video 1).

**Figure 2:**
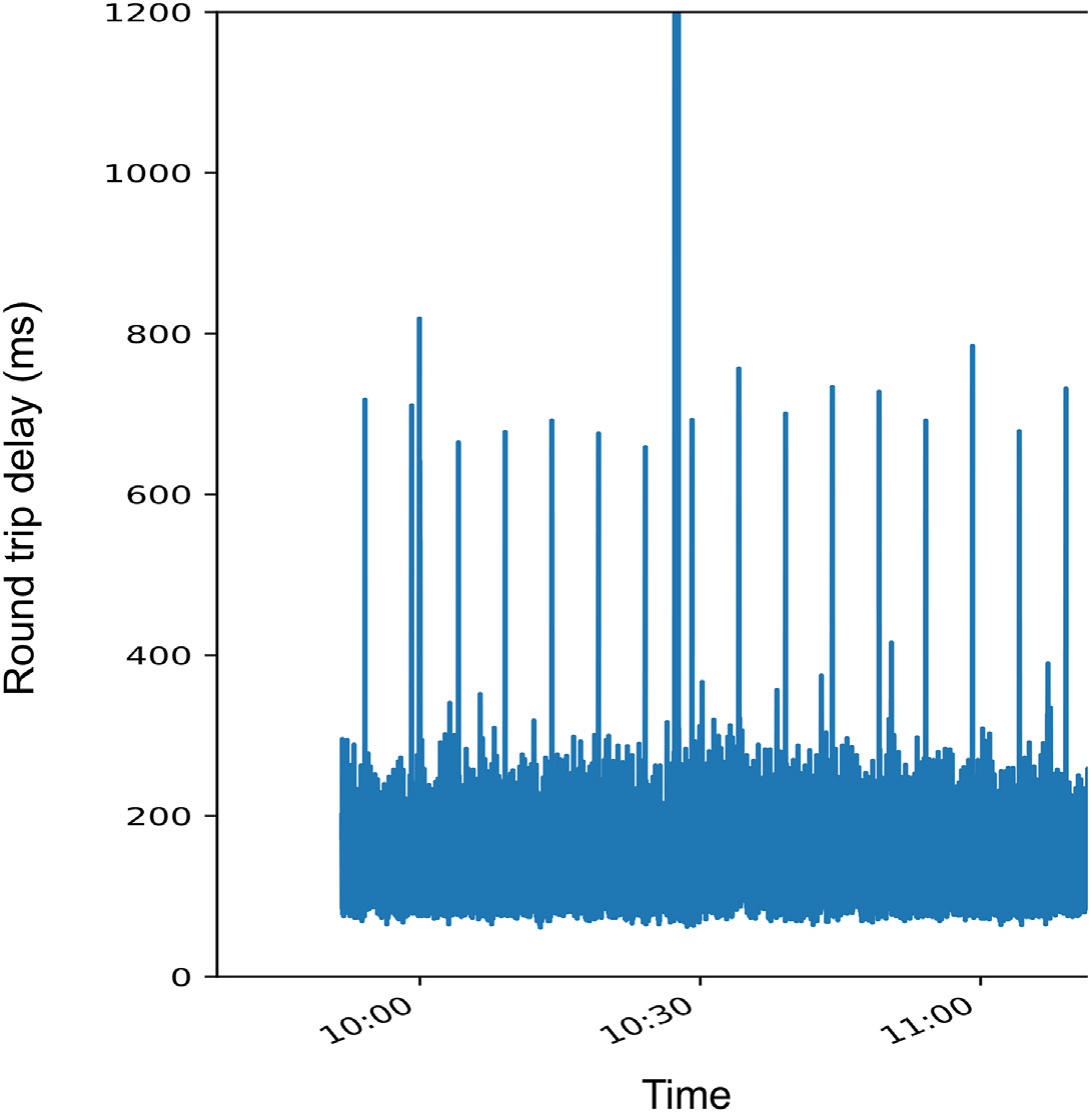
Graph showing communication delays.

The operation time was 2 hours 44 minutes, with minimal blood loss and no obvious organ damage. The operation time was measured in terms of console time, which was the time from the actual onsite robot docking to the undocking. The graph in Figure 2 shows the delay in communication, indicating that a delay of a few seconds of image disturbance or a delay of the operation occurred every 5 minutes. The measured delay is the pure communication delay, including the cloud virtual private network. The average round-trip latency was approximately 130 milliseconds. The surgeon and assistant communicated using the videoconference software Zoom (Zoom Video Communications, Inc). A different internet connection was used for the video conference, and no problems were encountered.

## Comment

Telesurgery is the practice of performing surgery using robotic devices and cutting-edge telecommunications technology by a surgeon who is physically distant from the patient. Several surgical specialties, such as urology, cardiology, and neurosurgery,^6^ have used telesurgery, proving its viability and potential to provide access to specialists, particularly in underserved areas. However, several challenges remain, including technical issues, cost, legal and regulatory frameworks, cybersecurity, and the need for a robust telecommunication infrastructure. Our study focused on technical issues and cost. Latency times and costs have improved since the Lindbergh operation in 2001, and we successfully reduced the latency time while keeping costs low in this experiment.

Technical issues are a primary concern, particularly network latency, bandwidth limitations, and safety. In this experiment, 2 types of communication delays were observed. One was the short delay in image transmission/reception and robot operation signals that occurred continuously throughout the experiment. The other was a delay of a few seconds that occurred every 5 minutes. The reason for the communication delays every 5 minutes might be satellite switching. Increased latency can notably impair surgical performance, with acceptable communication delays generally less than 100-320 milliseconds. Bandwidth fluctuations can also affect the quality of transmitted images and the responsiveness of robotic instruments.^7^^,^^8^ In experiments using geostationary earth orbit satellites, data latency was 600 milliseconds, making it difficult to perform the operation.^3^ Moreover, Takahashi and colleagues^9^ reported that a communication delay of 100-150 milliseconds indicated that surgery was not feasible. Although the communication delay should be less than 100 milliseconds according to the guidelines of the Japan Surgical Society,^10^ an anterior segmentectomy could be safely performed in a swine located 1000 km away, and the delay time of 130 milliseconds was acceptable. Delay elimination and stable communications are needed to improve the quality of surgery, and our engineers anticipate further improvements.

**Figure 3:**
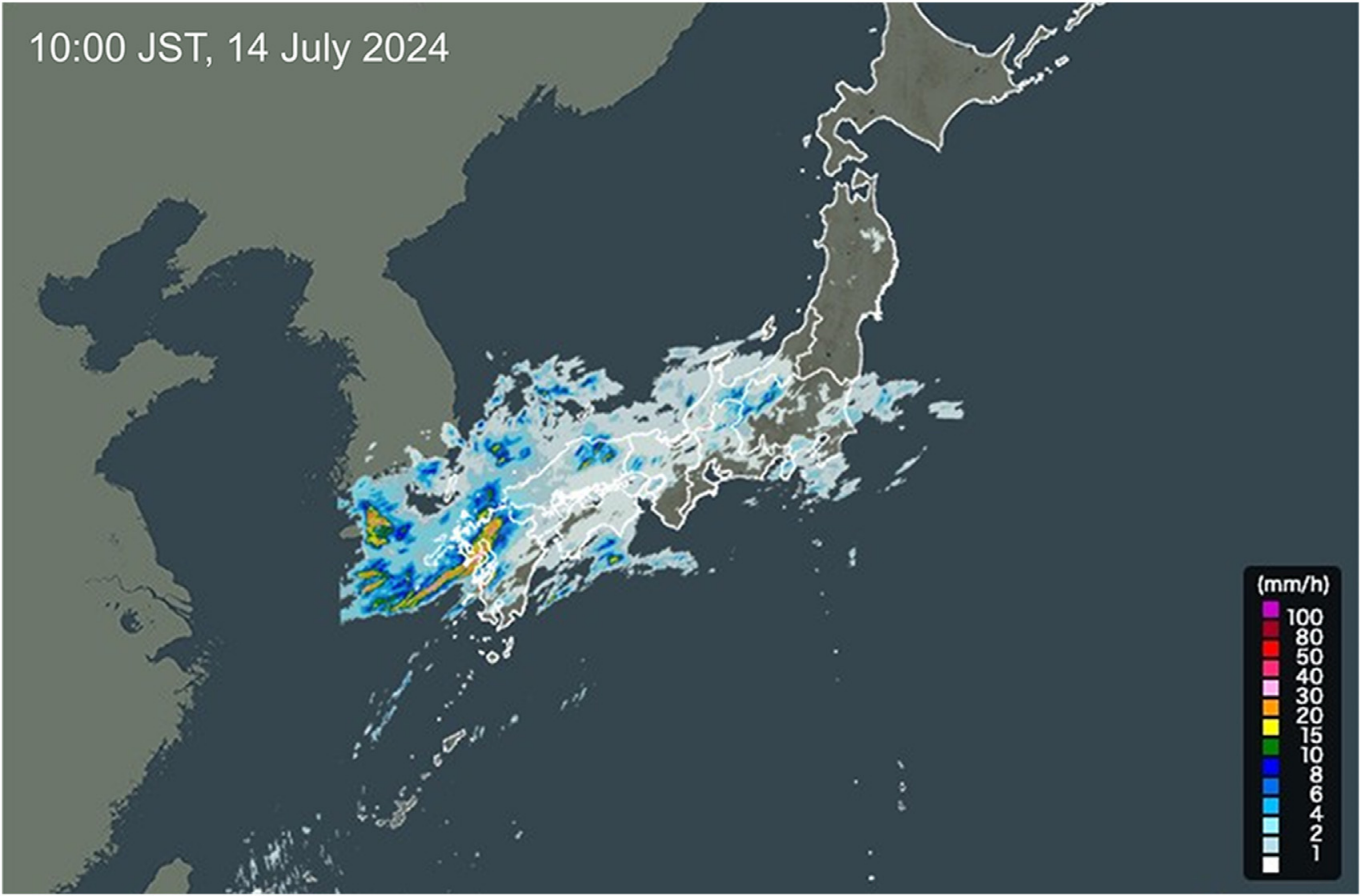
Color-coded precipitation information at the time of the experiment obtained from the Japan Meteorological Agency. Note the torrential rain in Fukuoka at the time of the experiment.

There are several reasons for communication delay. The communication speed of Starlink depends on the amount of cloud cover. Localized torrential rain occurred in Fukuoka on the experiment day (Figure 3), which might have been the main cause of the communication delay. Other causes of delay were the virtual private network server’s processing time, the robot’s internal processing, and image compression and decompression. The accumulation of these delays resulted in the average delay time. The safety of telesurgery should also be discussed. Robotic-assisted telesurgery has unique problems, including lack of tactile sensation and management of emergency situations. In addition to minimizing communication delays, it is important to use experienced physicians, who can act as assistants at the patient’s side in emergency situations to improve safety, and to establish a technology that can transmit tactile sensations to the surgeon. Saroa is a novel clinically applied surgical robot to provide haptic force feedback, and the tactile feedback function from a remote location was confirmed in this experiment.

Communication facilities and cost are other major issues. The communication facilities used in previous telesurgery experiments, such as submarine cables and dedicated 5G lines, take time to prepare and incur enormous costs. By comparison, Starlink requires no special installation and users can connect to the Starlink network using a small dish antenna and modem. The business model costs only US$180 per month. The total communication cost for this experiment was approximately US$6220.

In conclusion, we successfully performed remote robotic surgery from Fukuoka in Fukushima using Saroa and Starlink. The main problems related to telesurgery include technical limitations (communication facilities, latency, and bandwidth) and cost. The present study resulted in safe and cost-effective telesurgery. Challenges including legal and regulatory frameworks and cybersecurity should be addressed in the future.

## Freedom of Investigation

The tested technology was purchased. All authors had freedom of investigation and full control of the study design, methods, outcome parameters, analyses, and production of the article.

## Disclaimer

The Society of Thoracic Surgeons, The Southern Thoracic Surgical Association, and *Annals of Thoracic Surgery Short Reports* neither endorse nor discourage the use of the new technology described in this article.
